# Crack-Detection Algorithm Integrating Multi-Scale Information Gain with Global–Local Tight–Loose Coupling

**DOI:** 10.3390/e27020165

**Published:** 2025-02-05

**Authors:** Yun Bai, Zhiyao Li, Runqi Liu, Jiayi Feng, Biao Li

**Affiliations:** School of Aviation, Inner Mongolia University of Technology, Hohhot 010021, China; 20231800821@imut.edu.cn (Z.L.); 20231100541@imut.edu.cn (R.L.); 20241100548@imut.edu.cn (J.F.); 20230000036@imut.edu.cn (B.L.)

**Keywords:** crack detection, multi-scale information gain, mutual information optimization, conditional entropy minimization

## Abstract

In this study, an improved target-detection model based on information theory is proposed to address the difficulties of crack-detection tasks, such as slender target shapes, blurred boundaries, and complex backgrounds. By introducing a multi-scale information gain mechanism and a global–local feature coupling strategy, the model has significantly improved feature extraction and expression capabilities. Experimental results show that, on a single-crack dataset, the model’s mAP@50 and mAP@50-95 are 1.6% and 0.8% higher than the baseline model RT-DETR, respectively; on a multi-crack dataset, these two indicators are improved by 1.2% and 1.0%, respectively. The proposed method shows good robustness and detection accuracy in complex scenarios, providing new ideas and technical support for in-depth research in the field of crack detection.

## 1. Introduction

With the rapid development of deep learning technology, object detection has made significant progress in the field of computer vision and has been widely used in fields such as autonomous driving, intelligent monitoring, and infrastructure maintenance. Among them, crack detection, as an important task in infrastructure safety assessment, has received widespread attention. Efficient and accurate crack detection can significantly improve the maintenance efficiency of infrastructure and reduce potential safety hazards, while having important social and economic value.

However, existing object-detection algorithms still face numerous critical challenges in crack-detection tasks [1]. Cracks often appear as slender and irregularly shaped structures with diverse sizes and orientations, which significantly complicates feature extraction. Additionally, crack images typically have complex backgrounds, such as textures or shadows on concrete surfaces, making the boundaries between cracks and their backgrounds easily blurred and increasing detection difficulty. Furthermore, the characteristics of cracks can vary significantly across different scenarios, and existing algorithms often exhibit insufficient generalization capabilities in cross-scenario applications, further limiting their practical effectiveness [2,3].

In response to the above problems, this paper proposes an improved model based on information theory, which optimizes feature expression and detection performance from the information perspective through the tight coupling of global information and the loose coupling mechanism of local information. During the design process, we chose RT-DETR as the backbone of the model. This choice is mainly based on its excellent multi-scale feature extraction capability and efficient end-to-end target detection framework. RT-DETR achieves a good balance between accuracy and speed through a hybrid encoder and query selection strategy, while significantly improving the detection performance in complex scenes, especially in handling multi-scale targets and small target detection. As a basic network, RT-DETR provides a powerful feature extraction capability for the model in this paper, and has good compatibility with the multi-scale information gain mechanism and global–local feature coupling mechanism proposed in this paper, so that it can better serve the needs of crack-detection tasks.

In crack detection, feature information usually includes high-level semantic information and detail information. In order to improve the model’s perception of information at different levels, this paper introduces a multi-scale information gain mechanism and a conditional entropy optimization strategy. The multi-scale information gain mechanism can adaptively capture crack features of different scales and maximize the mutual information between crack features and input images, thereby improving the detection ability of crack targets; the global feature tight coupling mechanism enhances the model’s ability to capture semantic information by maximizing the mutual information between global features and original features; the local feature loose coupling mechanism highlights the importance of crack detail areas by minimizing the conditional entropy of local features, and effectively reduces the interference of complex background on detection.

The innovations of this paper include the following:

(1) First, two datasets are constructed for the detection scenarios of multiple cracks and single cracks to verify the effectiveness of the algorithm in this paper and other algorithms.
(2) Second, a multi-scale information gain mechanism is proposed to improve the detection effect by adaptively capturing crack features of different scales.
(3) Third, the design of tight coupling of global information and loose coupling of local information is combined to enhance the model’s perception of global semantic information and local detail information of cracks. From the perspective of information theory, the model in this paper can better solve the problems of blurred boundaries, background interference, and insufficient small target detection performance in crack detection in complex scenarios.

## 2. Related Work

### 2.1. Object Detection

As one of the core tasks in the field of computer vision, target detection aims to identify and locate targets in images. It is of great significance in scenarios such as autonomous driving [4] and security monitoring [5]. Two-stage detector [6,7] is one of the classic methods of target detection. This type of method first generates candidate areas and then classifies and regresses the candidate areas to finally achieve target detection. Typical representatives include Faster R-CNN [8], which greatly improves the efficiency of candidate region generation by introducing a region proposal network. Compared with traditional methods, it shows significant advantages in accuracy. However, since the two-stage method needs to complete candidate region generation and classification regression operations in sequence, it has certain limitations in real-time performance [9].

In contrast, the single-stage detector [10] directly models the target detection task as a regression and classification problem, abandons the candidate region generation process, and pursues a balance between speed and efficiency. For example, the Yolo [11] series achieves end-to-end efficient detection by simultaneously predicting the category and location of targets in a single network. Its innovation lies in simplifying the detection process into a regression problem, which greatly improves the detection speed, making it a representative method for real-time target detection; in addition, the SSD [12] model architecture predicts the target position by introducing multi-scale feature maps and categories, further improving the ability to detect small targets. Its lightweight architecture design enables it to adapt to application scenarios with strong real-time requirements while maintaining high accuracy.

In a recent study, Song et al. [13] proposed a single-stage directional object-detection method (SOD) based on ring heatmap and multi-stage angle prediction. The ring heatmap with multi-stage classification prediction angle and spatial constraints solved the boundary problem of targets with large aspect ratio and arbitrary orientation and verified its effectiveness and competitiveness on multiple challenging datasets. Ma et al. [14] proposed a task-coordinated single-stage object detector (TSOD), which improves the consistency and collaboration of classification and localization tasks by introducing task-decoupled feature alignment mechanism (TFAM) and task interaction enhancement mechanism (TEM). Due to its superior performance and real-time nature, currently, the single-stage target-detection model has become a popular research direction in industry and academia [15,16,17].

### 2.2. Object Detection Based on Transformer Architecture

In recent years, the Transformer [18] architecture has achieved remarkable success in the field of natural language processing and has gradually been applied to computer vision tasks, especially object detection, which has attracted widespread attention and research. The core advantage of Transformer lies in its global modeling ability, which can effectively capture long-distance dependencies in images, thus breaking through the limitations of traditional convolutional neural networks in receptive field.

DETR [19] is the first object-detection method based on the Transformer architecture, proposed by the Facebook AI team in 2020. This model directly introduces the Transformer into the object detection task, abandoning the hand-designed components in traditional detectors (such as anchor boxes and non-maximum suppression). DETR regards object detection as a set prediction problem in an end-to-end manner. Through the Transformer structure, a global attention mechanism is established between image features and queries, realizing the direct prediction of object categories and locations. Although DETR has shown strong expressive power in theory, its convergence speed is slow, especially in the detection performance of small objects and complex scenes; this aspect needs to be improved [20].

In view of the shortcomings of DETR, Deformable DETR [21] solves the problems of slow convergence speed and limited spatial resolution of DETR by introducing a deformable attention mechanism, achieving more efficient training and better detection performance for small targets; Dynamic DETR [22] solves the problems of slow convergence of DETR training and low resolution of small features by introducing a dynamic attention mechanism, replacing global attention with convolutional dynamic attention in the encoder and replacing cross attention with ROI-based dynamic attention in the decoder, greatly improving training efficiency and detection performance; at the same time, Up-DETR [23] enhances the feature learning ability of DETR through unsupervised pre-training methods, while optimizing multi-task learning and multi-query positioning, greatly improving training convergence speed and detection accuracy. In recent years, a series of improved models based on the Transformer architecture have emerged, such as Conditional DETR [24] and Anchor DETR [25], which optimize the structure and performance of DETR from the perspectives of query initialization and anchor design, respectively; and in the recent study, RT-DETR [26] improves the processing speed of multi-scale features by designing an efficient hybrid encoder, and introduces uncertainty minimization query selection to improve the accuracy of the decoder, achieving a breakthrough in the speed and accuracy of end-to-end object detection, reaching 53.1% AP and 108 FPS on the COCO dataset, surpassing the performance of the YOLO series and DINO. These studies have further promoted the application of Transformer in the field of object detection and provided new possibilities for more efficient and accurate object detection.

### 2.3. Crack Detection Based on Deep Learning

As an important task in infrastructure maintenance, crack detection has received extensive attention from deep learning in recent years. Deep-learning-based methods break through the limitations of traditional methods that rely on manually designed features through automated feature extraction and perform well in complex scenarios. Many researchers have proposed different innovative methods in this field, which have promoted the accuracy and robustness of crack detection [27,28,29].

Su et al. [30] proposed the MOD-YOLO algorithm, which improved the YOLO architecture through improved depthwise separable convolution, global receptive field spatial pyramid pooling, and coordinate attention, and improved channel information retention and multi-scale perception capabilities for efficient and accurate crack detection. Zhou et al. [31] proposed the ICDW-YOLO algorithm, which improved the neck network, layer structure and anchor algorithm of the YOLOv8 model, introduced a double-layer attention mechanism and dynamic gradient gain characteristics, and enhanced multi-scale feature fusion and small target recognition capabilities for efficient crack detection.

In addition to algorithm improvement, Narges Karimi et al. [32] used the YOLOv5 architecture combined with transfer learning and target-detection models to develop a deep-learning-based crack-detection method for crack identification in different cultural heritage materials and to achieve cross-material performance comparison. Qiu et al. [33] proposed a real-time crack-detection method based on the YOLO algorithm and drone platform. By optimizing and comparing multiple YOLO network architectures, they achieved fast and efficient tile crack detection and adapted to various environmental conditions.

These studies show that crack-detection methods based on deep learning have obvious advantages in handling complex scenes and improving detection accuracy, and have laid a technical foundation for the practical application of industrial crack detection in the future.

## 3. Method

**Figure 1:**
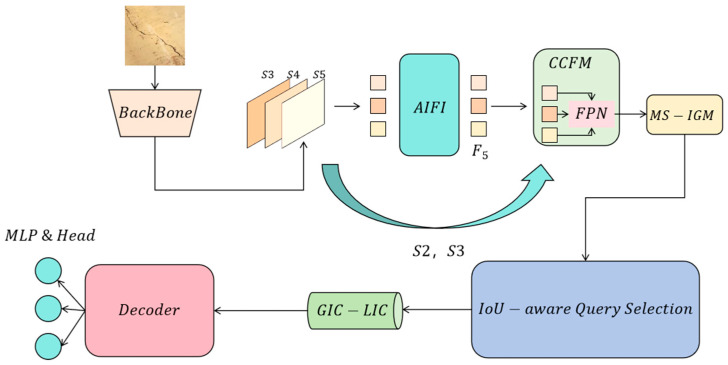
This figure shows the overall architecture of the improved crack-detection model, which is designed based on information theory and mainly includes feature extraction (backbone), multi-scale information gain module (MS-IGM), global–local information coupling module (GIC-LIC), query selection based on information entropy perception, and decoding process, forming an information-efficient detection process.

In the field of crack detection, due to slenderness, irregularity, and multi-scale complexity of cracks, as well as interference from the complex backgrounds of cracks, crack-detection tasks always face huge challenges. In order to improve the performance of the existing RT-DETR model in crack-detection tasks, this paper proposes an improved design based on multi-scale information gain optimization and tight coupling of global information and loose coupling of local information from the perspective of information theory. The overall model architecture is shown in Figure 1.

In order to further understand the algorithm flow proposed in this paper, and in order to determine how to optimize features in the multi-scale information gain module and the global–local information coupling module, this paper will analyze the multi-scale feature-generation process of the backbone network from the perspective of information theory, derive the local information optimization mechanism of MS-IGM, and the global information guided feature fusion method of GIC-LIC, and fully demonstrate the information processing and optimization process.

### 3.1. Multi-Scale Information Gain Module

Suppose the input image is $I\;\in \;R^{H\times W\times 3}$, where H and W are the height and width of the image, and 3 is the number of RGB channels. After the image is input, the backbone network is first used to extract features:

(1) $$F=BackBone(I),\;F\in R^{C\times H^{\prime }\times W^{\prime }}$$

where $H^{\prime }$ and $W^{\prime }$ are the width and height of the feature map after downsampling, and C is the number of channels. The backbone network usually contains convolutional layers, pooling layers, and nonlinear activation functions, and its specific form can be expressed as:

(2) $$F_{l}=\phi (W_{l}*F_{l-1}+b_{l}),\;l=1,2,\ldots ,L$$

where $F_{l}\;$represents the output of the l layer of the backbone network, $W_{l}$ and $b_{l}$ are the convolution kernel weight and bias, respectively, ∗ represents the convolution operation, and ϕ(·) is the activation function. The final output of feature extraction is a multi-scale feature map:

(3) $${\left\{F_{s}\right\}}_{s=1}^{S},\;F_{s}\in R^{C_{s}\times H_{s}\times W_{s}}$$

In the multi-scale feature map$,\;F_{s}$, each feature map,$\;F_{S}$, captures local information by defining a local neighborhood N(p), and the neighborhood is defined as:

(4) $$N(p)=\{p+△p\;|\;△p\in {\left\{-r,\ldots ,r\right\}}^{2}\},\;p\in F_{s}$$

where N(p) represents the neighborhood set of feature points at position p, and △p is the offset relative to p. The neighborhood range is determined by the radius r; that is, in two-dimensional space, the value range of r is ${\left\{-r,\ldots ,r\right\}}^{2}$. This means that the neighborhood contains all points whose Manhattan distance to p does not exceed r. The feature point p must belong to a specific feature map set $F_{s}$.

On this basis, in order to quantify the importance of feature points to crack detection in the neighborhood, the module introduces the information gain measurement mechanism. By calculating the conditional information gain (CIG) in the neighborhood, each feature point is given a weight. The weight calculation formula is:

(5) $$\alpha_{p}=\frac{exp(IG(q_{p},k_{q}))}{\sum_{j\in N(p)}exp(IG(q_{j},k_{q})}$$

where the information gain (IG) is defined as:

(6) IG(q,k)=H(q)−H(q|k)

where H(q) is the self-information entropy of the feature point, and H(q|k) is the feature entropy under the condition of given neighborhood features, which is specifically expressed as the uncertainty reduction in crack detection.

In the formula, $q_{k}=W_{q}F_{s}(q)$, $k_{p}=W_{k}F_{s}(p)$. $W_{q}$ and $W_{k}$ are mapping matrices used to map the original features to the query space and key space, so as to accurately model the correlation between different feature points in the high-dimensional space.

**Figure 2:**
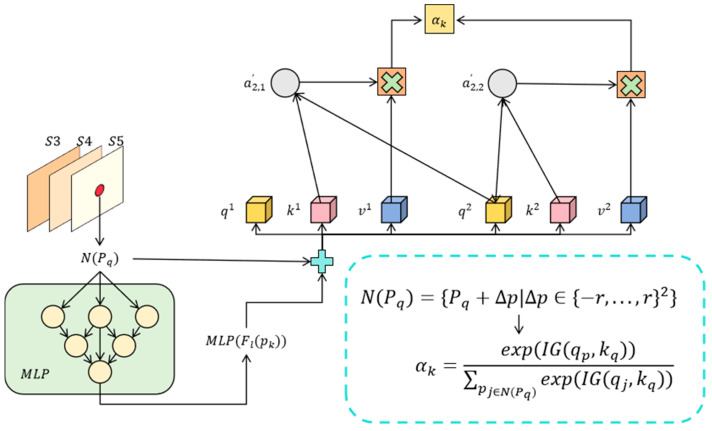
This figure shows the local feature extraction and weighting process of MS-IGM, which realizes dynamic feature fusion through neighborhood definition and attention weights.

After introducing information gain optimization, the module not only improves the sensitivity of the model to the relationship between features, but also enhances the ability to capture crack details by quantifying the effectiveness of neighborhood features; thereby, it adapts to the detection needs of complex backgrounds and multi-scale targets. The overall structure of the module is shown in Figure 2.

Furthermore, the weight $\alpha_{q}$ is used to weight the neighborhood features and integrate them to obtain the local feature $z_{p}$.

(7) $$z_{p}=\sum_{p\in N(p)}\alpha_{p}\cdot F_{s}(q)$$

In each layer of feature maps, the local features,$\;z_{p}$, of all points are combined into a new feature map, ${\bar{F}}_{s}$, and further multi-scale fusion is used to generate the final representation:

(8) $${\bar{F}}_{s}(p)=\sum_{s=1}^{S}w_{s}\cdot {\bar{F}}_{s}(p)$$

This process achieves effective integration of multi-scale features by dynamically allocating attention weights within the neighborhood. By introducing linear projection operations of queries and keys, the module can adaptively capture the global correlation and local dependency between feature points, thereby effectively distinguishing the target area from background noise in complex scenes. At the same time, the multi-scale fusion strategy further enhances the model’s sensitivity to target features of different sizes and shapes, especially when dealing with slender and irregular cracks. This design not only improves the accuracy of feature expression, but also enhances the robustness and generalization ability of the model in complex detection tasks.

### 3.2. Global–Local Information Coupling

Furthermore, in order to enhance the expressive power of global features and optimize the distribution of local features, this paper proposes a feature optimization method based on information theory that combines tight coupling of global information with loose coupling of local information. In this method, the mutual information of global features is maximized to enhance the representativeness of global information, while the conditional entropy of local features is minimized to improve the expression of local features in crack-detection tasks. First, we extract global features, and the formula is shown in Equation (8).

(9) $$F_{g}=G(F)=W_{g}\cdot GlobalAvgPool({\bar{F}}_{s})$$

where $W_{g}$ is the mapping matrix of the global feature, and GlobalAvgPool represents the global average pooling operation, which is used to remove the local imbalance features in the channel dimension and obtain the global feature $F_{g}\in R^{C}$.

Next, in order to enhance the expressive power of global features, the mutual information optimization objective is introduced:

(10) $$G^{*}={argmax}_{G}I(G;{\bar{F}}_{s})-\lambda \cdot H(G)$$

where $I(G;{\bar{F}}_{s})$ represents the mutual information between the global feature and the input feature, H(G) is the entropy of the global feature, and λ is the balance coefficient, which is used to weigh the amount of information and feature compactness. By maximizing the mutual information, it is ensured that the global feature contains more information related to the crack-detection task while reducing redundancy.

**Figure 3:**
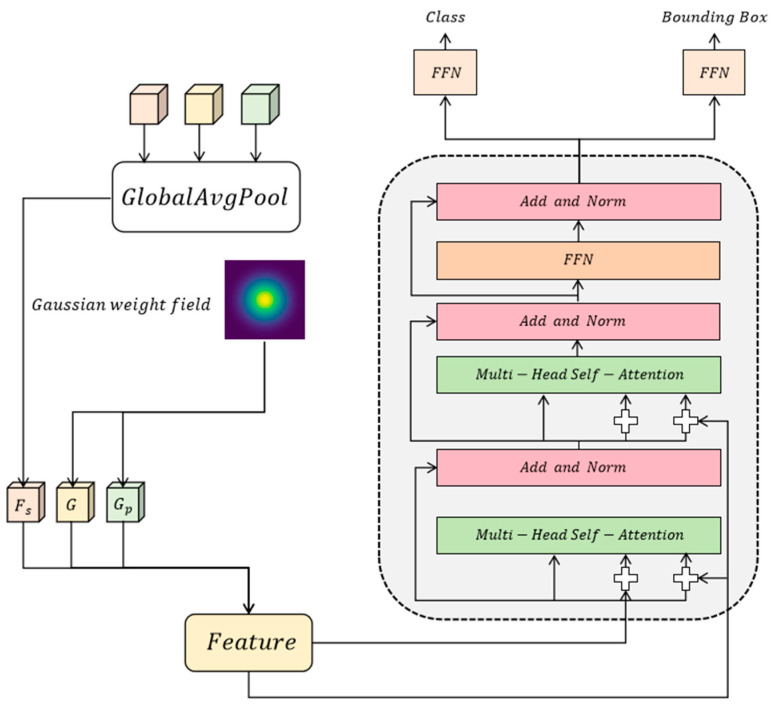
This figure shows the architecture of the feature processing module, which combines global average pooling and Gaussian weighted fields to achieve tight and loose coupling operations between global and local features, and output classification and bounding box prediction results. The module further enhances the feature expression capability and improves detection performance through a multi-head self-attention mechanism.

After optimizing the global features, a loose coupling mechanism for local features is introduced. Local feature loose coupling optimizes feature distribution based on the conditional entropy minimization objective. Formulas (11) and (12) describe the optimization process of local features:

(11) $$L_{p}={\bar{F}}_{s}(p)+exp(-\frac{{\left||{\bar{F}}_{s}(p)-G_{p}|\right|}^{2}}{2\sigma^{2}})$$

(12) $$L_{p}^{*}={argmin}_{L_{p}}H(L_{p}|G_{p})$$

where $L_{p}\;$is the local feature, $G_{p}$ is the projection of the global feature at position p, and σ is the smoothing parameter of the Gaussian distribution, which is used to control the looseness of the feature. The Gaussian weighting mechanism can dynamically adjust the balance between global and local features, and effectively enhance the model’s sensitivity to crack details by assigning higher weights to the significant areas of local features. At the same time, the background noise is significantly weakened by the attenuation factor of the Gaussian distribution, thereby improving the ability to distinguish local features. On this basis, the expression of local features is further optimized by the conditional entropy minimization method, making the feature distribution more robust and able to adapt to complex background environments. Ultimately, this mechanism not only enhances the saliency of the crack area, but also ensures the reasonable interaction between global information and local features. Its module architecture is shown in Figure 3.

In order to achieve the combination of tight coupling of global features and loose coupling of local features, the optimized final feature A is defined as:

(13) $$F^{*}=\alpha \cdot G^{*}+(1-\alpha )\cdot L^{*}$$

where A is the weight coefficient, which is used to balance the coupling degree between global features and local features. Through this coupling design, global features provide semantic information of cracks, while local features enhance the ability to capture crack details, thereby improving the accuracy and robustness of the detection task.

The final optimized feature, $F^{*}$, is the input into the decoder, D, and further interacts with the decoder state, S, to generate the final output:

(14) $$Y=softmax(W_{o}\cdot D(F^{*}\cdot S))$$

where $W_{o}$ is the output weight matrix, and the softmax operation is used to normalize the output to ensure the stability of the output. The above method effectively retains the global semantic information of the cracks and enhances the analysis ability of the detail area through the mutual information and conditional entropy optimization of global features and local features.

By combining the design of tight coupling of global features and loose coupling of local features, the feature-optimization module proposed in this paper significantly improves the overall performance and robustness in the crack-detection task and effectively solves the problems of blurred target boundaries and background interference. At the same time, this method does not need to introduce a large number of additional parameters or complex feature operations, maintains a low computational complexity, and is suitable for real-time scenarios.

## 4. Data Collection and Annotation

The data collection part includes the construction of single-crack datasets and complex multi-crack datasets. The data sources are mainly divided into two parts: one is to obtain crack images from public datasets, research databases, and open resources in related fields through network searches to ensure the diversity and representativeness of the data; the other is to collect crack images through offline field photography, using digital cameras and smartphones to obtain high-quality crack samples in different environments. The collected images include both simple scenes of single cracks and complex scenes with multiple cracks, aiming to build a dataset covering a variety of actual situations.

**Figure 4:**
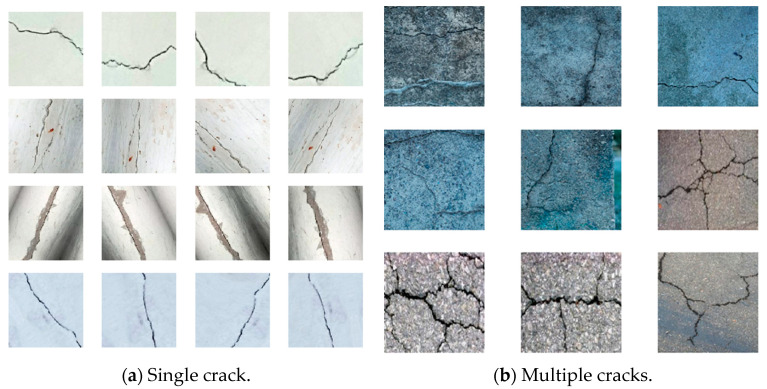
This figure shows examples of our datasets. (**a**) Examples from our single road crack dataset; (**b**) examples from our multiple road crack datasets.

In the data annotation process, the X-AnyLabeling tool was used to annotate the collected crack images. The annotation process follows strict specifications to ensure the accuracy and consistency of the annotation results, providing reliable basic data for the training and evaluation of subsequent models. Samples from the dataset are shown in Figure 4 as examples.

## 5. Experiment

This section aims to verify the effectiveness and robustness of the proposed model and analyze the performance of the model through a series of experiments. The experimental design is divided into four parts: first, through comparative experiments, the performance of the proposed model is compared with the current mainstream methods to evaluate the superiority of the model; second, hyperparameter sensitivity experiments are carried out to analyze the impact of key hyperparameters on model performance; third, ablation experiments are carried out to explore the contribution of each module to the overall performance by removing or adjusting different modules of the model; finally, the prediction results and feature extraction capabilities of the model are intuitively displayed through visualization experiments, verifying the actual application effect of the model in crack-detection tasks from multiple dimensions.

### 5.1. Experimental Details

During the experiment, this paper uses the same hyperparameter settings as RT-DETR. Specifically, the feature extractor selects ResNet50 pre-trained on ImageNet. The AdamW optimizer is used during training, the initial learning rate is 1 × 10^−4^, the cosine annealing learning rate scheduling strategy is adopted, the weight decay coefficient is 0.05, and the batch size is set to 16. The total number of training rounds is 50 rounds, and data enhancement methods such as random horizontal flipping and random cropping are used to improve the generalization ability of the model. In addition, to avoid gradient explosion, the gradient clipping threshold is set to 0.1, and the EMA (exponential moving average) strategy is added during the model training process to further stabilize the model performance. All experiments are conducted under NVIDIA 4090D. The specific hardware and software configurations of the experiment are shown in Table 1.

### 5.2. Performance Comparison Experiment

In the comparative experiments, in order to comprehensively evaluate the performance of the proposed model, it was compared with a variety of current mainstream target-detection models. The selected comparative models include the classic target detection algorithms SSD [12], YOLOV5 [34], YOLOV8 [35], Faster R-CNN [8], and the latest version of the baseline model YOLOV10 [36], which are widely used in industrial scenarios. In addition, this paper also compares with the latest research results R-CNN (RGA and PRM) [37], MOD-YOLO [30], DINO [38], DQ-DETR [39], KD-DETR [40], and the baseline RT-DETR [26] used in this paper. These models cover different detection frameworks and methods, including both lightweight and fast detection models and high-performance models that focus on accuracy. Through the comparison, the superiority and adaptability of the proposed model in crack-detection tasks can be fully verified.

As can be seen from Table 2, the improved model proposed in this paper performs well in the single crack-detection task. Compared with the baseline model RT-DETR, the improved model improves by 1.6% and 0.8% in the two key indicators of mAP@50 and mAP@50-95, respectively. This performance improvement is due to the introduction of the multi-scale information gain mechanism and the global–local feature coupling design, which optimizes the extraction and expression capabilities of crack features from the perspective of information theory. Especially in the single crack scene with a long crack length and irregular shape but a relatively simple background, the model effectively improves the positioning and classification accuracy of the crack area by maximizing the mutual information between the crack feature information and the global information, while reducing the conditional entropy of the background interference information, and maintains a low computational complexity, providing an efficient solution for the actual deployment of crack detection.

**Figure 5:**
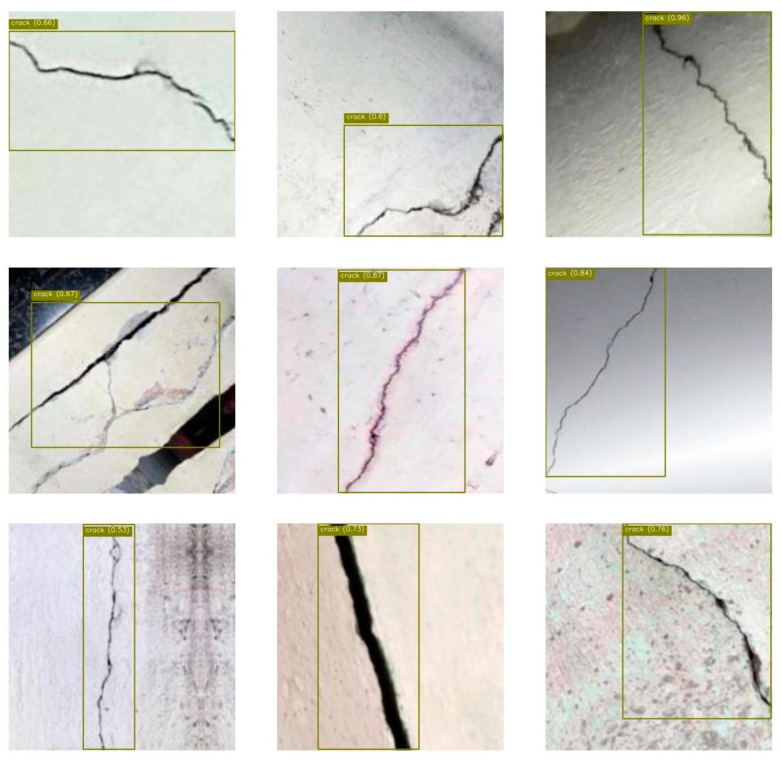
This figure shows the intuitive visualization results of our model on a single-crack dataset.

**Figure 6:**
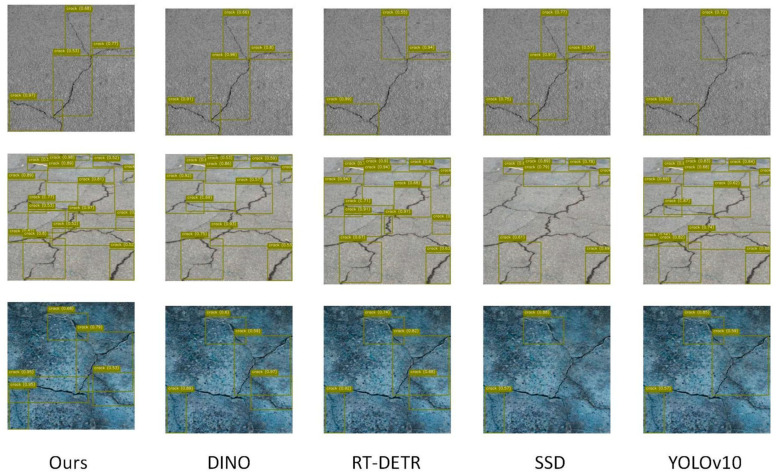
This figure shows a visual comparison of our model with other models.

In addition, in complex multi-crack scenes (as shown in Table 3), the proposed model still shows strong robustness and detection capabilities. Compared with mainstream detection algorithms, the performance of the proposed model in mAP@50 and mAP@50-95 is in a leading position, reaching 56.8% and 26.3%, respectively. This improvement is attributed to the reasonable coupling design of the model for global and local features, which enables it to effectively distinguish the boundaries and adjacent areas of multiple cracks under complex background interference. At the same time, the multi-scale information gain mechanism optimizes the detection ability of small cracks and overlapping cracks by dynamically adjusting the weights of crack features, thereby further improving the overall performance of the model. Experimental results show that the model not only performs well in simple scenarios but also can meet the high-precision requirements of crack detection in complex practical environments. In addition to the quantitative analysis, this paper also shows the intuitive experimental visualization results of the model in Figure 5 and Figure 6, which further verifies the effectiveness of this method.

### 5.3. Hyperparameter Sensitivity Experiments

After conducting comparative experiments, this paper further explores the hyperparameters of the article in order to gain a deeper understanding of the role of each module of the model and its performance in different scenarios. The main goal of the hyperparameter experiment is to reveal how key parameters affect the model’s stability, convergence speed, and ability to adapt to different crack complexities, providing data support and theoretical basis for subsequent model improvement designs.

First, this paper explores the impact of common hyperparameters. The experiment focuses on learning rate and optimizer. The experimental results are shown in Table 4 and Table 5.

From the experimental results in Table 4 and Table 5, it can be seen that the learning rate and optimizer have a certain impact on the model performance. In the learning rate experiment, as the learning rate is gradually reduced from 0.005 to 0.001, and the recall and mAP indicators show a stable improvement; mAP@50-95 especially reaches a high value of 25.7 at a learning rate of 0.001. When the learning rate is further reduced to 0.0005, the model performance continues to improve slightly, indicating that a smaller learning rate is conducive to the convergence and performance optimization of the model. In the optimizer experiment, the AdamW optimizer achieved the best results, with a recall of 47.6. mAP@50 and mAP@50-95 reached 56.8 and 26.3, respectively; these results are significantly better than those of AdaGrad and SGD, reflecting the advantages of AdamW in stability and optimization ability. This shows that the reasonable choice of learning rate and the use of optimizer have an important impact on the final performance of the model.

### 5.4. Data Preprocessing Experiment

In the crack-detection task, data preprocessing has an important impact on model performance. In order to evaluate the effects of different data augmentation strategies, this experiment designed multiple sets of comparative experiments, including only using basic preprocessing, a single augmentation strategy, and a comprehensive solution combining multiple augmentation strategies. By comparing the performance of these preprocessing methods in terms of recall, mAP@50, and mAP@50-95 indicators, we analyzed the role of data augmentation in improving the model’s generalization ability, robustness, and detection accuracy, thereby providing effective support for the optimization of the crack-detection model. The experimental results are shown in Table 6.

It can be seen from the experimental results that different data preprocessing methods have a significant impact on the performance of the model; the gradual superposition of preprocessing strategies and the recall, mAP@50, and mAP@50-95 indicators of the model all show a trend of gradual improvement. Basic preprocessing provides the basic guarantee for model training, but its performance is relatively limited; a single enhancement strategy can further improve the model’s sensitivity to detail features and slightly improve the detection performance. When two different enhancement methods are combined, the model performance is further improved, especially showing better robustness in complex backgrounds; and when all preprocessing strategies are combined, the model achieves the best effect, indicating that these enhancement methods complement each other at different levels and jointly improve the model’s ability to express crack features and detection accuracy. This result proves that the reasonable selection and combination of data preprocessing methods can not only alleviate the problem of uneven data distribution, but also significantly improve the model’s adaptability and generalization ability to diverse crack scenarios.

### 5.5. Ablation Experiment

In order to verify the contribution of each module of the model to the overall performance, this paper designed an ablation experiment to conduct a comparative analysis by gradually removing or replacing key modules. The experiment mainly focuses on the multi-scale information gain module (MS-IGM) and the global feature tight coupling and local feature loose coupling module (GIC-LIC), and evaluates their impact on model accuracy and robustness in different scenarios. By removing the MS-IGM module, the experiment reveals the important role of multi-scale information gain in improving the expression ability of crack features. It effectively captures multi-scale crack information and enhances the detection ability of small cracks by maximizing the mutual information between crack features and input images. By removing or replacing the GIC-LIC module, the contribution of global feature mutual information optimization and local feature conditional entropy constraint is further analyzed, verifying the key role of capturing global semantic information and highlighting local detail features in improving detection performance under complex backgrounds. By comparing with the complete model, the ablation experiment clearly reveals the specific role of each module in optimizing crack detection performance.

As can be seen from Table 7, the modules proposed in this paper improve the model performance to varying degrees. Based on the basic model RT-DETR, after adding the multi-scale information gain module (MS-IGM), the mAP@50-95 of the model increased by 0.3%, indicating that the module effectively enhances the ability to capture local features by maximizing the mutual information between crack features and input features. When the global feature tight coupling and local feature loose coupling module (GIC-LIC) is added, the recall and mAP@50-95 of the model are increased by 0.3% and 0.5%, respectively, indicating that the module significantly improves the detection accuracy of the model in complex backgrounds by optimizing the mutual information of global features and minimizing the conditional entropy of local features. Finally, when all improved modules work together, all indicators of the model reach the optimal level: recall is increased to 47.6%, and mAP@50 and mAP@50-95 are increased to 56.8% and 26.3%, respectively, which fully verifies the overall effectiveness of the improved design in this paper.

### 5.6. Visualization Experiment

Finally, we also give two parts of visual experimental results. One of the experimental results is the changes in the loss function and evaluation index of a single-crack dataset during the single dataset training process, and the other experimental result is the related feature map of the features.

**Figure 7:**
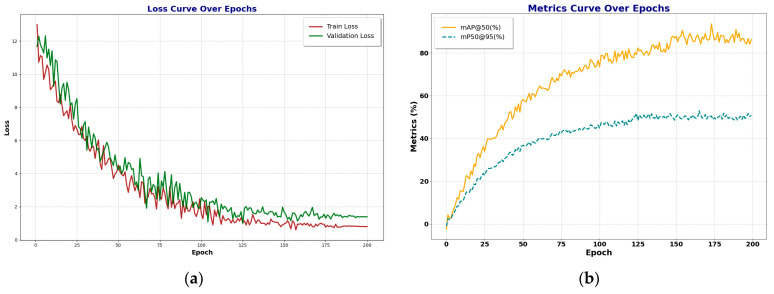
This figure shows the relevant images during the training process. (**a**) The loss function decreases with epoch; (**b**) The evaluation value increases with the epoch.

As can be seen from Figure 7, the model shows good convergence and stability during the training process. The loss curve on the left shows that both the training loss and the validation loss gradually decrease with the increase in training rounds and tend to be stable in the later stage, indicating that the model effectively reduces the error and does not overfit during the continuous optimization process. The mAP curve on the right shows that the mAP@50 and mAP@50-95 indicators gradually increase with the increase in rounds and tend to saturate in the later stage of training, reaching higher stable values. This shows that the model can continuously improve its detection accuracy as the training deepens, verifying the effectiveness and robustness of the proposed method.

**Figure 8:**
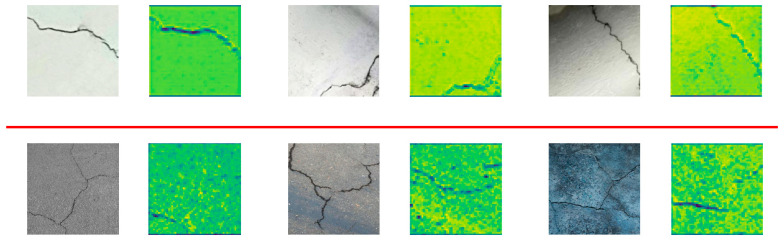
Feature maps of single-crack images and multi-crack images.

**Figure 9:**
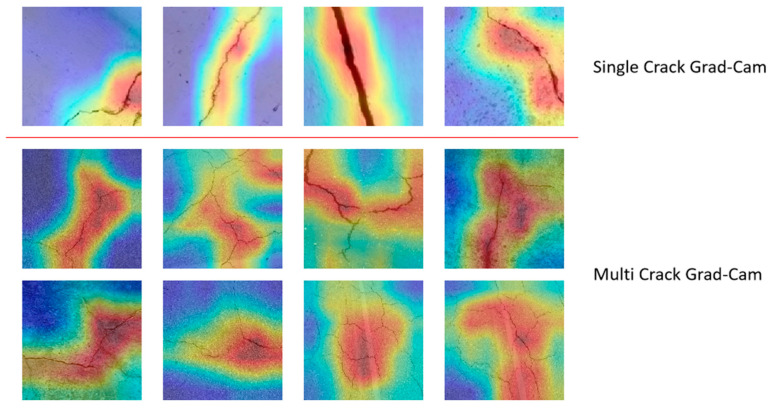
Grad-Cam experimental results on two different datasets.

As shown in Figure 8, it can be concluded that the model shows strong feature extraction capabilities when dealing with single-crack- and multiple-crack-detection tasks. The upper part shows the detection results of a single crack. The model can accurately capture the boundary features of the crack, especially when there is less background interference. By maximizing the mutual information of the crack area, the feature information of the crack area can be effectively highlighted. The lower part shows the detection results of multiple cracks. Even in a complex background, the model can still effectively separate the crack features by optimizing the conditional entropy of the crack features and accurately locate and characterize the crack area. Overall, this verifies the robustness and accuracy of the multi-scale information gain mechanism and global–local feature coupling design proposed in this paper in different scenarios. In order to further verify the effect of the model, this paper also uses Grad-Cam to perform visual analysis on two different datasets. The experimental results are shown in Figure 9.

## 6. Practical Implications and Conclusions

### 6.1. Practical Significance

Finally, we explored the potential value of our method in practical applications. Crack detection is crucial in infrastructure maintenance, especially in large-scale industrial scenarios and complex environments. Efficient and accurate detection methods can significantly improve maintenance efficiency and reduce potential safety hazards. The improved model proposed in this paper not only improves the accuracy of crack detection by optimizing multi-scale information gain and global–local information coupling design but also shows excellent robustness and generalization ability in complex backgrounds and multi-crack scenarios. This performance improvement provides technical support for the real-time monitoring and maintenance of infrastructure such as bridges and roads and lays the foundation for intelligent detection systems in large-scale industrial applications.

### 6.2. Conclusions

This paper proposes an improved crack-detection model that combines multi-scale information gain and global–local feature coupling to deal with the problems of multi-scale targets and complex background interference in crack-detection tasks. By maximizing the mutual information of global features and minimizing the conditional entropy of local features, the model has achieved significant improvements in feature expression ability and detection performance. Experimental results show that the mAP@50 and mAP@50-95 of the model on a single-crack dataset reached 85.8% and 47.8%, respectively, and the mAP@50 and mAP@50-95 on a multi-crack dataset reached 56.8% and 26.3%, respectively, which are significantly better than the baseline model RT-DETR and other mainstream detection algorithms. Future work will focus on model lightweighting and real-time detection capability optimization to meet the practical application needs of crack detection in large-scale industrial environments.

## Figures and Tables

**Table 1:** Specific experimental parameter configuration table.

Experimental Details	Specific Configuration
CPU	I7–14,700 kf
Operating System	Ubuntu22.04
Memory	16 GB
GPU	NVIDIA 4090D
Cuda	12.0
Python	3.10
Torch	2.3

**Table 2:** Experimental results on a single-crack dataset.

Model	Recall	mAP@50	mAP@50-95	FLOPs	Params
SSD	60.2	59.1	12.4	32.7	8.2 M
YOLOv5-M	75.6	68.4	33.2	64.2	25.1 M
YOLOV8-M	76.3	69.5	34.5	78.9	25.9 M
Faster R-CNN	77.5	71.3	36.4	334.5	136.6 M
YOLOV10-M	78.4	72.7	35.7	59.1	15.4 M
R-CNN (RGA and PRM)	79.2	74.1	39.2	324.6	134.5 M
MOD-YOLO	82.1	77.3	39.8	60.3	7.17 M
DINO	83.1	81.6	43.7	311	66 M
RT-DETR	85.1	84.0	47.0	136	42 M
DQ-DETR	85.1	84.0	47.2	263	72 M
KD-DETR	85.2	84.8	48.1	245	65 M
Ours	85.8	85.6	47.8	147	43.1 M

**Table 3:** Experimental results on a multi-crack dataset.

Model	Recall	mAP@50	mAP@50-95	FLOPs	Params
SSD	21.4	32.7	15.1	32.7	8.2 M
YOLOv5	22.8	34.2	16.3	64.2	25.1 M
YOLOV8	31.9	39.3	17.4	78.9	25.9 M
Faster R-CNN	34.9	42.7	19.8	334.5	136.6 M
YOLOV10	34.3	42.5	17.7	59.1	15.4 M
R-CNN (RGA and PRM)	38.1	46.5	21.7	324.6	134.5 M
MOD-YOLO	36.7	44.8	19.2	60.3	7.17 M
DINO	39.1	48.2	22.6	311	66 M
RT-DETR	46.6	55.6	25.3	136	42 M
DQ-DETR	46.8	55.8	25.9	263	72 M
KD-DETR	47.5	56.5	26.1	245	65 M
Ours	47.6	56.8	26.3	147	43.1 M

**Table 4:** Different experimental results brought by different learning rates.

Lr	Recall	mAP@50	mAP@50-95
0.005	46.4	55.3	25.1
0.003	46.7	55.6	25.4
0.002	46.6	55.5	25.1
0.001	46.8	55.9	25.7
0.0005	47.6	56.8	26.3

**Table 5:** Different experimental results from different optimizers.

Optimizer	Recall	mAP@50	mAP@50-95
AdaGrad	43.2	52.1	23.1
SGD	45.6	54.5	24.8
Adam	47.1	55.9	26.1
AdamW	47.6	56.8	26.3

**Table 6:** Comparative experimental results of different data preprocessing.

Different Preprocessing Methods	Recall	mAP@50	mAP@50-95
Basic preprocessing	45.2	55.1	25.3
+Histogram Equalization (HE)	46.5	55.5	25.5
+Random horizontal flip	46.8	55.9	25.7
+Random mask	46.8	55.9	25.7
+HE + Random horizontal flip	47.1	56.3	26.0
+HE + Random mask	47.2	56.4	26.1
+Random horizontal flip + Random mask	47.4	56.6	26.2
+All	47.6	56.8	26.3

**Table 7:** Ablation experiment results.

Model Setup	Recall	mAP@50	mAP@50-95
RT-DETR	46.6	55.6	25.3
+MS-IGM	47.1	56.2	25.6
+GCC-LIC	46.9	56.1	25.8
+ALL	47.6	56.8	26.3

## Data Availability

The data presented in this study are available on request from the corresponding author.
